# Insomnia and progression to total joint replacement in hip (41 737) and knee pain (81 958): a prospective UK biobank cohort study

**DOI:** 10.1136/rmdopen-2025-006357

**Published:** 2026-01-16

**Authors:** Travis Haber, Brett Dyer, David M Klyne, Paulo H Ferreira, Philippa J A Nicolson, Belinda J Lawford, Susan M McCurry, Michelle Hall

**Affiliations:** 1 Centre for Health, Exercise and Sports Medicine, Department of Physiotherapy, The University of Melbourne, Melbourne, Victoria, Australia; 2 Griffith Biostatistics Unit, Griffith Health, Griffith University, Gold Coast, Queensland, Australia; 3 Centre for Innovation in Pain and Health Research (CIPHeR), School of Health Sciences, The University of Queensland, Brisbane, Queensland, Australia; 4 Musculoskeletal Research Hub, Charles Perkins Centre, The University of Sydney, Sydney, New South Wales, Australia; 5 Physiotherapy Research Unit, Oxford University Hospital NHS Foundation Trust Nuffield Orthopaedic Centre, Oxford, Oxfordshire, UK; 6 Nuffield Department of Orthopaedics, Rheumatology and Musculoskeletal Sciences (NDORMS), University of Oxford, Oxford, Oxfordshire, UK; 7 School of Nursing, Department of Child, Family, and Population Health, University of Washington, Seattle, Washington, USA; 8 Sydney Musculoskeletal Health, The Kolling Institute, The University of Sydney, Sydney, New South Wales, Australia

**Keywords:** Osteoarthritis, Rehabilitation, Osteoarthritis, Knee

## Abstract

**Objective:**

Insomnia often co-exists with hip or knee pain and is associated with greater pain severity. However, there is limited evidence on whether insomnia contributes to progression to joint replacement. Using data from the UK Biobank, we tested whether symptoms of insomnia among people with hip or knee pain are associated with undergoing total hip or knee joint replacement surgery.

**Methods:**

UK Biobank data from participants with hip (n=41 737) or knee pain (n=81 958) in the past 3 months were included. Using self-reported baseline data, participants were classified as ‘never’, ‘sometimes’ or ‘usually’ having insomnia symptoms (ie, trouble falling asleep or waking in the night). We examined associations between baseline symptoms of insomnia and undergoing total hip or knee replacement surgery using adjusted Cox proportional hazards models.

**Results:**

In knee pain, ‘usually’ experiencing insomnia symptoms was associated with undergoing total knee replacement (adjusted HR 1.14 (95% CI 1.04 to 1.25)), within, but not beyond, 4.7 years of enrolment, compared with ‘never’ experiencing insomnia symptoms. No association was observed for ‘sometimes’ experiencing insomnia symptoms and total knee replacement among individuals with knee pain, nor for insomnia symptoms (‘usual’ or ‘sometimes’) and total hip replacement among individuals with hip pain.

**Conclusion:**

Insomnia may be a modifiable factor contributing to earlier progression to knee replacement. Targeting insomnia through interventions could form part of a holistic approach to managing chronic knee pain. Further research is needed to determine whether managing insomnia can reduce the risk of knee replacement surgery.

**WHAT IS ALREADY KNOWN ON THIS TOPIC**

- Individuals with painful hip or knee osteoarthritis with co-existing insomnia often experience worse pain severity, which may hasten the progression to total joint replacement surgery.

**WHAT THIS STUDY ADDS**

- Frequent insomnia is a potentially modifiable factor that may contribute to early progression to knee replacement for knee pain, but no association was observed between insomnia and hip replacement.

**HOW THIS STUDY MIGHT AFFECT RESEARCH, PRACTICE OR POLICY**

- Managing insomnia in individuals with knee osteoarthritis may be a strategy to help slow progression to knee replacement, but randomised controlled trials are needed to evaluate whether insomnia is causally related to illness progression in this patient population.

## Introduction

 Pain is the primary symptom of osteoarthritis, often leading to difficulties with walking, daily functioning and social participation.^1 2^ Persistent pain, despite having tried recommended nonsurgical treatments such as exercise,^3 4^ is a primary factor influencing the decision to undergo hip or knee joint replacement.^5^ Compared to 2020, cases of knee and hip osteoarthritis are projected to increase by about 75% and 79%, respectively, by 2050,^6^ placing increasing strain on healthcare systems.^7 8^ Reducing the need for joint replacement requires a more complete understanding of the factors associated with disease progression. Although pain remains central, research has largely focused on pathoanatomical and biomechanical drivers of pain.^9^ Other potentially modifiable factors remain underexplored, despite emerging evidence that various factors, such as insomnia, may exacerbate joint symptoms^10 11^ and could therefore contribute to progression to joint replacement.

Insomnia is characterised by difficulty initiating sleep, maintaining sleep or early-morning awakening despite adequate opportunity and circumstances for sleep.^12^ Insomnia commonly causes clinically significant distress or impairment in social, occupational or other important areas of functioning.^12^ Although definitions for insomnia are inconsistent, up to 63% of those with symptomatic hip and knee osteoarthritis have insomnia.^13^ While there is a possible bidirectional relationship between sleep and pain,^14^ growing evidence suggests that sleep disturbances may exacerbate joint pain severity among people with osteoarthritis.^15^,^18^ For example, among individuals with knee pain and multisite pain from the longitudinal Tasmanian Older Adult Cohort, worse sleep disturbances were associated with greater pain severity, and these associations were maintained up to 10.7 years after enrolment.^19^ Of note, cognitive behavioural therapy is the first-line core treatment for insomnia, which modestly improves osteoarthritis-related pain.^20 21^ This provides evidence for a relationship between sleep disturbances, such as insomnia, and pain severity associated with osteoarthritis.^22^ By influencing joint pain, sleep quality may be a modifiable factor influencing progression towards joint replacement.

15 17

Only one prior study has assessed whether insomnia is associated with joint replacement among people with osteoarthritis.^23^ It reported no association, but its follow-up period was relatively short (3 years), and participants were not restricted to those with hip or knee osteoarthritis (ie, it also included types of osteoarthritis that do not lead to hip or knee joint replacement surgery).^23^ To the best of our knowledge, no long-term studies have examined the association between insomnia and the incidence of joint replacement surgery for hip or knee osteoarthritis. Using the large, longitudinal UK Biobank Cohort, we tested whether symptoms of insomnia among people with hip or knee pain are associated with undergoing total hip or knee joint replacement surgery. We hypothesised that symptoms of insomnia would be associated with the incidence of total joint replacement among individuals with hip or knee pain.

## Methods

### Study design and participants

**Figure 1:**
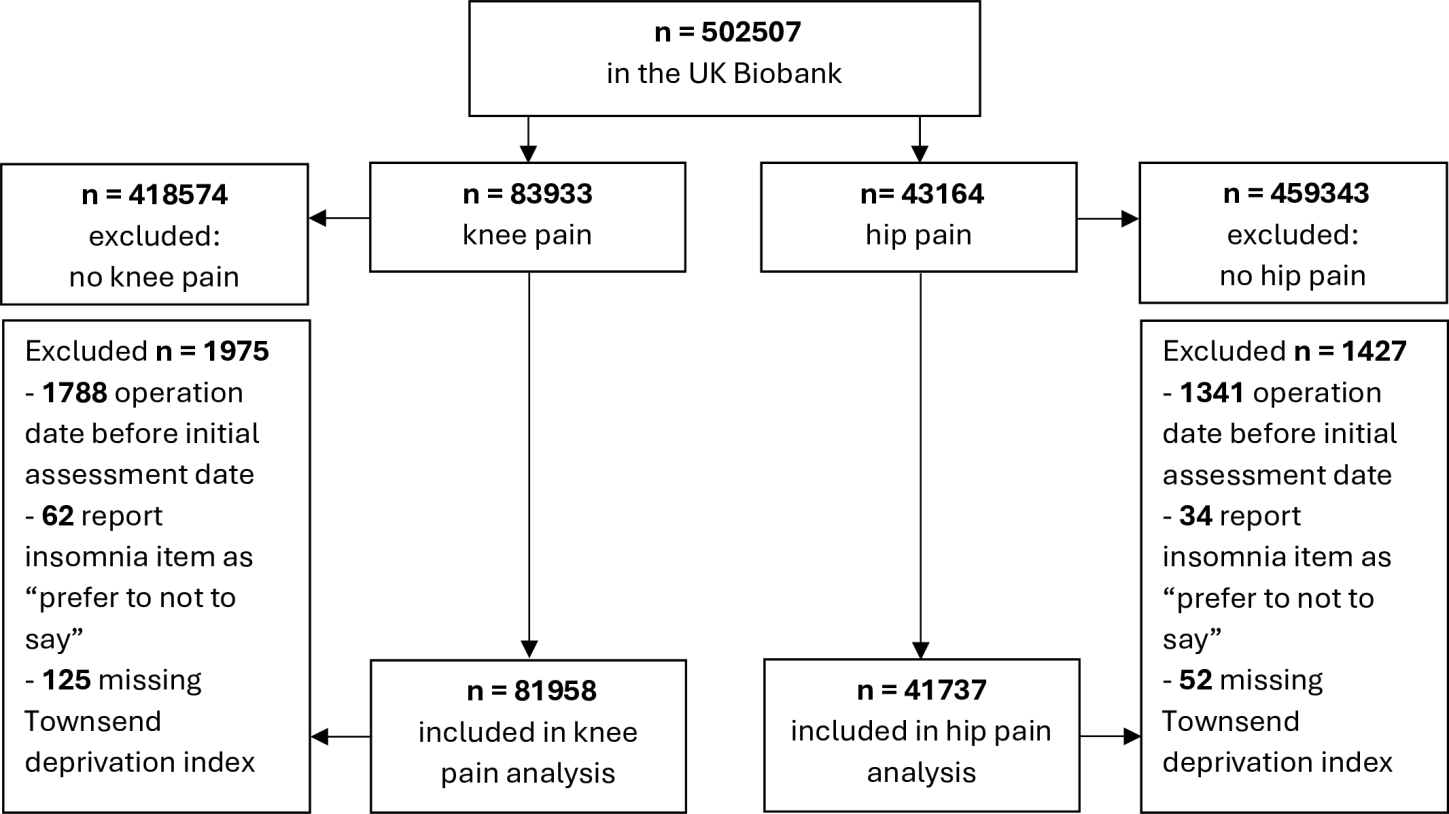
Flow diagram of participant inclusion. Participants self-reported knee or hip pain in the past 3 months at baseline inclusion to the UK Biobank.

This study was reported according to the Strengthening the Reporting of Observational Studies in Epidemiology guidelines for observational studies.^24^ We used data from the UK Biobank—a databank which first enrolled a cohort of 502 507 individuals from the community aged 40–69 years (age group chosen to include people at risk of developing long-term conditions), enrolled between 2006 and 2010.^25^ The UK Biobank collected comprehensive data on participants’ sociodemographic characteristics, medical history and health behaviours at baseline. Follow-up data are included in the UK Biobank, partly through linkage with other UK national datasets. Linked data sets for hospital data are as follows: Inpatient hospital admission data for England comes from the Hospital Episode Statistics Admitted Patient Case data set; inpatient hospital data for Wales comes from the Patient Episode Database for Wales Admitted Patient Care; and inpatient data for Scotland comes from two datasets: the General Acute Inpatient and Day Case - Scottish Morbidity Record (SMR01) and the Mental Health Inpatient and Day Case - Scottish Morbidity Record (SMR04). We included participants in this study who indicated either hip and/or knee pain in the past 3 months (Data-Field 3773 and Data-Field 3414, respectively). We extracted separate datasets for participants reporting knee or hip pain to perform analysis for hip and knee pain separately, noting that 18 666 participants were included in both hip and knee analyses. We excluded participants who had their first total hip or knee replacement surgery before baseline and participants with missing covariate data (125 participants (<1%) with missing data for knee pain and 52 participants (<1%) for hip pain) (figure 1).

### Exposure

Information about insomnia was collected in the initial assessment period (at the Assessment Centre, via touchscreen questionnaire) with the following question (Data Field 1200): ‘‘Do you have trouble falling asleep at night or do you wake up in the middle of the night?’ If participants selected the help button, they were shown the following message: ‘If this varies a lot, answer the question in relation to the last 4 weeks.’ Participants could respond (1) never, (2) sometimes, (3) usually or (4) prefer not to say. Those participants responding with ‘prefer not to say’ were excluded from the study (63 for knee pain (<1%) and 34 for hip pain (<1%)).

### Outcome

We identified the events of total hip replacements and knee replacements using Operating Procedure Codes Supplement (OPCS) Classification of Interventions and Procedures 4 (OPCS-4) (data-coding 240) and OPCS3 (data-coding 259), included in the UK Biobank via linked inpatient hospital data (as described above), which denote specific medical procedures and operations undertaken by the National Health Service (NHS), in the UK. Please see online supplemental Material(p.1) for codes of surgical procedures used to identify knee and hip replacements in the UK. We identified these codes through a previous study^26^ and in consultation with a clinician-researcher (PJAN) with expert knowledge in this area. We did not consider the time to partial or unicompartmental joint replacements, or to revision joint replacement surgeries in the same participant.

### Observation period

The observation period in this study started from the initial assessment period (ie, the date at which participants were enrolled in the UK Biobank, Data-Field 53) until the date of one of the following, depending on which came first: total hip or knee replacement, death or the end of hospital inpatient data collection (see: https://biobank.ndph.ox.ac.uk/showcase/exinfo.cgi?src=Data_providers_and_dates). Data on UK Biobank participants who died come from the linked data sets: for England and Wales, the NHS England, and for Scotland, the NHS Central Register, a part of the National Records of Scotland. The end date of data collection varied depending on whether a person had inpatient data for England, Wales or Scotland. Given that most participants in the UK Biobank reside in England (89% were recruited in England for baseline assessment), for those participants without an inpatient code, the English data collection end date was used.

### Covariates

The following potential confounders were selected based on our expert field knowledge and available evidence^27^,^35^: sex, body mass index (height and weight measured during initial assessment centre visit), ethnicity (self-report during initial assessment visit), education (self-report during initial assessment visit), depression (over past 2 weeks, self-report during initial assessment visit) and Townsend Deprivation Index (a measure of material deprivation in an area, with greater scores indicating worse progression, calculated immediately before entering the UK Biobank based on national census data). The rationale for selecting these covariates is provided in online supplemental file 1. The Townsend Deprivation Index was used rather than the Index of Multiple Deprivation, as it is measured uniformly across the cohort, whereas the Index of Multiple Deprivation is calculated differently across England, Scotland and Wales. See figure 1 of the Supplementary Material for the directed acyclic graphs (DAGs) we created using Daggity (https://www.dagitty.net/dags.html),^36^ depicting potential confounding variables, as well as the potentially confounding variables for data that are not available in the UK Biobank or the linked datasets. For the covariate depression, participants answering ‘prefer not to answer’ (knee pain: 426 (1%); hip pain: 198 (1%)) and ‘do not know’ (knee pain: 4078 (5%); hip pain: 2054 (5%)) were not included in the relevant models. See table 1 of the online supplemental materials for categories created for the covariate variables for analyses.

### Data analysis

All data management and statistical analysis were performed in R (R Core Team 2024) using the packages ‘Survival’ and ‘Survminer’.^37^,^39^ Continuous data were presented as means (SD) or as medians (IQR), depending on data distribution. Categorical variables were presented as frequencies and percentages. Categorical data were presented as frequency counts and percentages. Cox proportional hazards models were used to estimate hazard ratios (HRs) for hip and knee replacement between those who reported they ‘sometimes’ or ‘usually’ experienced symptoms of insomnia and those who reported they ‘never’ experienced symptoms of insomnia (‘never’ being used as the reference category). The proportional hazards assumption was checked by plotting Kaplan-Meier curves and assessing Schoenfeld and log-log plots for both hip and knee pain models. In the knee pain model, there was evidence that the HR for knee replacement was different for the first 4.7 years compared with the period from 4.7 years from enrolment to the end of follow-up, thus violating the proportional hazard assumption. To address this, the Cox model was stratified into the following two time groups: (1) from the start of the observation period to 4.7 years and (2) from 4.7 years until the end of the observation period. Thus, separate HRs are presented for each of these two time periods in the knee pain model. There was no evidence to suggest a violation of the proportional hazards assumption in the hip pain model, so one HR is presented for the full follow-up period. Plots of Kaplan-Meier survival estimates for not undergoing knee replacement or hip replacement are presented.

### Sensitivity analysis

When constructing the DAG, there was uncertainty in the temporal ordering of depression and insomnia, because depression and insomnia may cause each other, but also because the coding may not reflect the exact point in time when the individual started experiencing depression/insomnia. To assess the sensitivity to misspecification of the adjustment set, each model was run once with the primary adjustment set (ie, sex, body mass index, ethnicity, education and Townsend Deprivation Index) and once with depression also accounted for. If both models produce similar results, then this provides reassurance that the results are not sensitive to the misspecification of the adjustment set.^40^

To assess the sensitivity of results to unmeasured confounding, we calculated E values for the HR point estimate and lower bound of the 95% CI.^41^ E values estimate the size of the association that an unmeasured confounder would need to have with both the exposure and the outcome to completely explain away the observed association.

We also performed a sensitivity analysis to ensure findings did not vary if we assumed that all people resided in England, compared with if we assumed all people without an inpatient code (thus making the residing country unknown) resided in Wales—the country with the most different end of data collection date from England’s, differing by approximately 5 months. Further, the Cox models were re-run, excluding participants with co-existing hip and knee pain, to assess the sensitivity of the results to changing the inclusion/exclusion criteria.

## Results

Figure 1 presents the flow diagram of participant inclusion. See table 1 for participant characteristics. A total of 81 958 participants with knee pain were included. Participants with knee pain were more likely to be female (54%) and were often overweight (41%) or obese (37%), educated to the level of a degree or professional qualification (40%) and most reported their ethnicity as British, Irish and/or white (94%). Of these participants, 45% reported ‘sometimes’ having insomnia, and 37% ‘usually’ having insomnia. Overall, 10 907 (13%) participants with knee pain underwent a total knee replacement, with a median time to event of 6.6 years (IQR 3.6–9.5). The median follow-up period for participants with knee pain (ie, from admission into the Biobank until the index date) was 13.6 years (IQR Q1–Q3, 12.7–14.3).

**Table 1:** Participant characteristics across those with knee or hip pain (reported as n (%) unless otherwise stated)

Characteristics	Knee pain (n=81 958)	Hip pain (n=41 737)
Age at recruitment (median (IQR (Q1–Q3))	59 (52–64)	60 (53–64)
Sex		
Female	44 285 (54)	26 726 (64)
Male	37 673 (46)	15 011 (36)
Body mass index (kg/m^2^)		
Underweight <18.5	209 (<1)	156 (<1)
Normal weight (18.5–24.9)	16 894 (21)	9616 (23)
Overweight (25–29.9)	33 743 (41)	16 684 (40)
Obese (>30)	30 598 (37)	14 968 (36)
Unknown	514 (<1)	313 (1)
Symptoms of insomnia		
Never	14 556 (18)	6076 (15)
Sometimes	36 918 (45)	17 816 (43)
Usually	30 484 (37)	17 845 (43)
Depression (past 2 weeks)		
Not at all	53 899 (66)	26 398 (63)
Several days	17 525 (21)	9595 (23)
More than half the days	3520 (4)	2006 (5)
Nearly every day	2510 (3)	1486 (4)
Do not know	4078 (5)	2054 (5)
Prefer not to answer	426 (1)	198 (1)
Educational status		
No degree or professional qualification	28 755 (35)	14 518 (35)
Degree or professional qualification	32 657 (40)	16 298 (39)
Prefer not to say or none of the above	19 936 (24)	10 601 (25)
Unknown	610 (1)	320 (1)
Townsend Deprivation Index (mean (SD)^†^	−0.960 (3)	−0.927(3)
Ethnicity		
British, Irish and/or white	76 715 (94)	39 714 (95)
Asian or of part Asian background	2185 (3)	736 (2)
African, black, Caribbean or with part black background	1758 (2)	739 (2)
Other ethnic group	985 (1)	404 (<1)
Do not know or prefer not to say	315 (<1)	144 (<1)
Had a total joint replacement (number of events (%))	10 907 (13)	5358 (13)
Time to joint replacement (years, median IQR (Q1–Q3))^‡^	6.6 (3.6–9.5)	5.3 (2.4–8.9)
Time to follow-up (years, median IQR (Q1–Q3))	13.6 (12.7–14.3)	13.6 (12.7–14.3)

* Percentages may not equal 100% due to rounding.

† Positive values indicate areas with high material deprivation, while negative values indicate relative affluence. A score of 0 represents an area with mean values.

‡ Calculated as median time to first knee or hip replacement from baseline assessment in UK Biobank.

Q1, quartile 1; Q3, quartile 3.

A total of 41 737 participants with hip pain were included. Participants with hip pain were more likely to be female (64%) and were often overweight (40%) or obese (36%), educated to the level of degree or professional qualification (39%), and most reported their ethnicity as British, Irish and/or white (95%). Of these participants, 43% reported symptoms consistent with insomnia ‘sometimes’ and 43% ‘usually’. Overall, 5358 (13%) participants with hip pain underwent a total hip replacement, with a median time to event of 5.3 years (IQR 2.4–8.9). The median follow-up period for participants with hip pain (ie, from admission into the Biobank until the index date) was 13.6 years (IQR Q1–Q3, 12.7–14.3).

**Figure 2:**
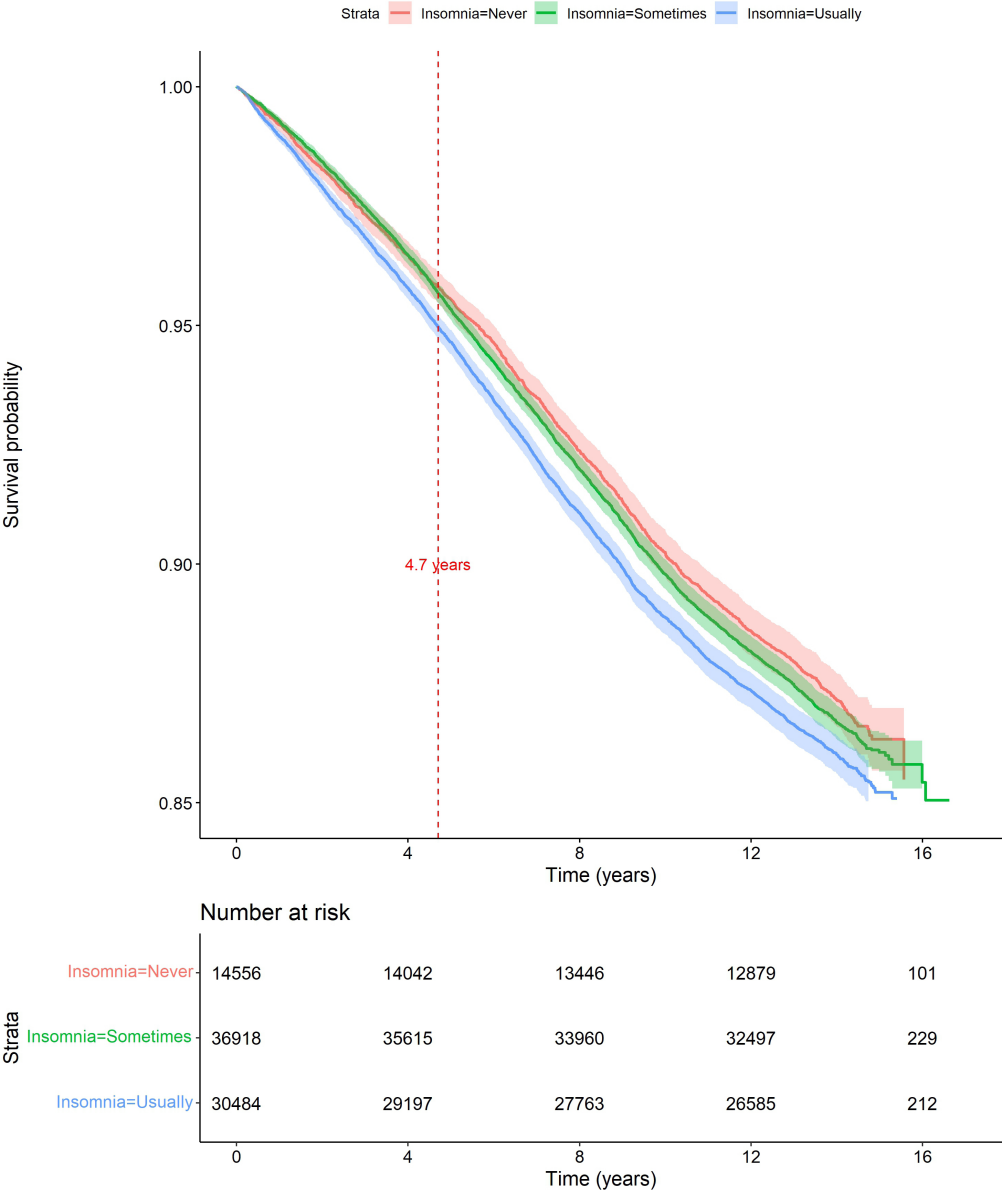
Plot of Kaplan-Meier estimates (with 95% CIs) for not undergoing total knee replacement, comparing participants by symptoms of insomnia.

Figure 2 shows Kaplan-Meier survival estimates for not undergoing total knee replacement. Unadjusted and adjusted results are presented in table 2. In the adjusted analysis, from enrolment to 4.7 years, experiencing ‘usual’ symptoms of insomnia was associated with undergoing total knee replacement (HR 1.14, 95% CI 1.04 to 1.25). There was no evidence of an adjusted association (HR 1.00, 95% CI 0.93 to 1.07) between ‘usually’ experiencing symptoms of insomnia and undergoing total knee replacement for the period after 4.7 years. No associations were observed between ‘sometimes’ experiencing symptoms of insomnia and undergoing total knee replacement for either the first 4.7 years (1.01, 95% CI 0.92 to 1.11) or the period after 4.7 years (1.02, 95% CI 0.95 to 1.08). Joint replacements by insomnia severity are presented in table 3.

**Table 2:** Unadjusted and adjusted associations between symptoms of insomnia and incident total joint replacement

	Unadjusted analysis			Adjusted analysis*		
	**Knee pain** (n=81 958)		**Hip pain** (n=41 737)	**Knee pain** (n=81 958)		**Hip pain** (n=41 737)
**Symptoms of insomnia**	Time period 1: <4.7 years HR (95% CI)	Time period 2: >4.7 years HR (95% CI)	HR (95% CI)	Time period 1: <4.7 years HR (95% CI)	Time period 2: >4.7 years HR (95% CI)	HR (95% CI)
Sometimes	1.03 (0.94 to 1.13)	1.04 (0.97 to 1.11)	0.98 (0.90 to 1.06)	1.01 (0.92 to 1.11)	1.02 (0.95 to 1.08)	0.98 (0.90 to 1.06)
Usual	1.21 (1.10 to 1.32)	1.05 (0.99 to 1.13)	0.93 (0.85 to 1.00)	1.14 (1.04 to 1.25)	1.00 (0.93 to 1.07)	0.94 (0.87 to 1.02)
Never	REF	REF	REF	REF	REF	REF

Unadjusted and adjusted Cox proportional hazards models estimating associations between sleeplessness and total knee replacement.

* Adjusted for sex, body mass index, ethnicity, education and Townsend Deprivation Index.

REF, reference.

**Table 3:** Joint replacements according to the severity of symptoms of insomnia

	Knee pain (n=81 958)	Hip pain (n=41 737)
**Symptoms of insomnia**		
Never	1846 (2%)	812 (2%)
From enrolment to 4.7 years	610 (1%)	
From beyond 4.7 years	1236 (2%)	
Sometimes	4834 (6%)	2328 (6%)
From enrolment to 4.7 years	1596 (2%)	
From beyond 4.7 years	3238 (4%)	
Usual	4227 (5%)	2218 (5%)
From enrolment to 4.7 years	1532 (2%)	
From beyond 4.7 years	2695 (3%)	

**Figure 3:**
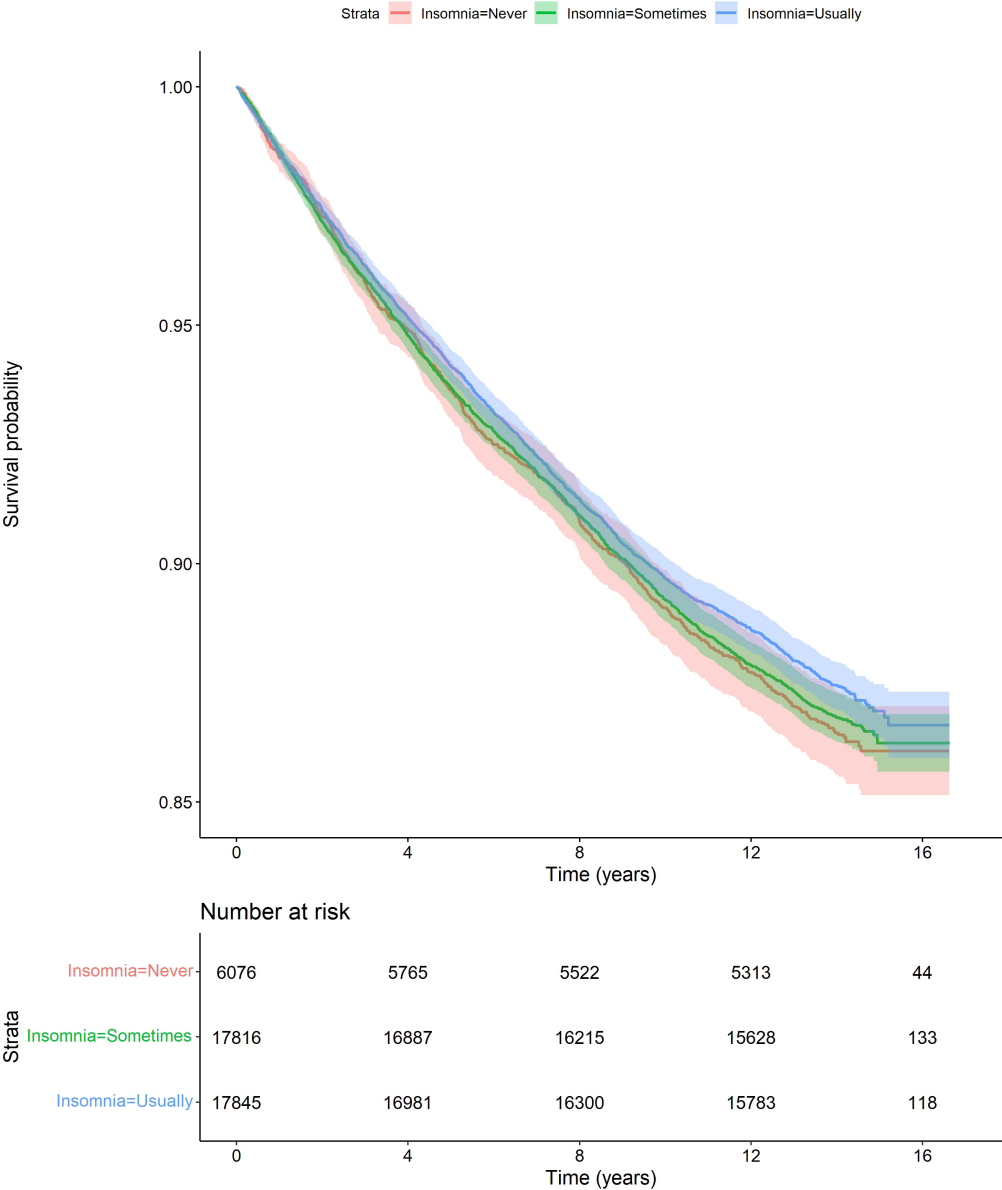
Plot of Kaplan-Meier estimates (with 95% CIs) for not undergoing total hip replacement, comparing participants by symptoms of insomnia.

Figure 3 shows Kaplan-Meier survival estimates for not undergoing total hip replacement. Unadjusted and adjusted results are presented in table 2. In the adjusted analysis, among those with hip pain, there was no association between ‘sometimes’ (HR=0.98, 95% CI 0.90 to 1.06) or ‘usually’ (HR=0.94, 95% CI 0.87 to 1.02) experiencing symptoms of insomnia and undergoing total hip replacement.

Associations between symptoms of insomnia and incident total knee replacement were similar in the sensitivity analysis that included an additional covariate, depression (see table 4). The E values for the HR point estimate and lower bound of the 95% CI for the association between usually experiencing symptoms of insomnia and undergoing total knee replacement (0–4.7 years) were 1.54 and 1.24 for the primary analysis. In the sensitivity analysis including depression, the E values were 1.67 and 1.37 (see figures2  3 of the Supplementary Material for the E plots). Thus, if there were a hypothetical unmeasured confounder that had an association of this magnitude with both the exposure and the outcome, then this could have explained away the observed associations (ie, primary analysis: HR 1.14; sensitivity analysis: HR 1.20). A sensitivity analysis showed no substantial differences in associations between symptoms of insomnia and incident total knee replacement when missing country codes were imputed as England compared with Wales (table 2 of the online supplemental material). Therefore, results were robust to how censoring dates were defined when inpatient codes were missing. Lastly, a sensitivity analysis showed no substantial differences in associations between symptoms of insomnia and incident total knee replacement when participants with co-existing hip and knee pain were excluded (see table 3 of online supplemental material).

**Table 4:** Sensitivity analysis: associations between symptoms of insomnia and incident total joint replacement, including an additional covariate of depression

Unadjusted analysis				Adjusted analysis*		
	**Knee pain** (n=77 454)		**Hip pain** (n=39 485)	**Knee pain** (n=77 454)		**Hip pain** (n=39 485)
**Symptoms of insomnia**	Time period 1: <4.7 years HR (95% CI)	Time period 2: >4.7 years HR (95% CI)	HR (95% CI)	Time period 1: <4.7 years HR (95% CI)	Time period 2: >4.7 years HR (95% CI)	HR (95% CI)
Sometimes	1.03 (0.93 to 1.13)	1.04 (0.97 to 1.11)	0.98 (0.90 to 1.06)	1.03 (0.93 to 1.13)	1.04 (0.97 to 1.11)	1.01 (0.93 to 1.09)
Usual	1.19 (1.08 to 1.30)	1.05 (0.98 to 1.13)	0.92 (0.84 to 0.99)	1.20 (1.09 to 1.32)	1.06 (0.99 to 1.14)	1.00 (0.92 to 1.09)
Never	REF	REF	REF	REF	REF	REF

Unadjusted and adjusted Cox proportional hazards models estimating associations between sleeplessness and total knee replacement.

* Adjusted for sex, body mass index, ethnicity, education, Townsend Deprivation Index and depression.

REF, reference.

## Discussion

This study investigated whether baseline insomnia was associated with undergoing total hip or knee replacement surgery in a large cohort of people with hip or knee pain. Among individuals with knee pain, those with ‘usual’ symptoms of insomnia had higher rates of total knee replacement over the first 4.7 years from study enrolment, but not beyond 4.7 years after being enrolled in the UK Biobank. In contrast, among individuals with hip pain, ‘usual’ symptoms of insomnia were not associated with a higher rate of undergoing total hip replacement. These findings suggest that frequent insomnia may contribute to progression toward total joint replacement in individuals with knee pain, but not in those with hip pain.

In people with knee pain, ‘usually’ experiencing symptoms of insomnia was only associated with undergoing total knee replacement within the first 4.7 years after enrolling in the UK Biobank. This observation partially supports our hypothesis and requires several considerations. First, it may reflect that insomnia exacerbates knee pain severity and related physical disability, thereby accelerating the decision to undergo knee replacement in the short term (relative to the time of UK Biobank enrolment). Mechanisms that might explain this relationship include the potential role of insomnia in altering both peripheral and central sensitisation, thereby up-regulating or down-regulating nociceptive processing.^42 43^ For example, insomnia may reduce activation of the endogenous analgesic systems, such as the endogenous opioid system.^22^ Insomnia has also been proposed to influence pain via its effects on inflammatory pathways (eg, upregulation of pro-inflammatory cytokines), alterations in functional neural connectivity across pain-related brain networks and its contribution to maladaptive pain cognitions such as pain catastrophising.^22 42 44 45^ Beyond 4.7 years, factors other than insomnia, such as the exhaustion of conservative treatment and advice from health professionals, may be more prominent or take precedence in the decision to undergo knee replacement.^46^ Second, the observation may indicate that people with usual symptoms of insomnia undergo knee replacement surgery earlier, resulting in a residual cohort with lower risk or delayed need for knee joint replacement. Third, as more knee joint replacements occurred after 4.7 years (65%) than before 4.7 years (35%), our statistical power was unlikely to be compromised in detecting an association after 4.7 years, if present.

These findings are partly consistent with a prior study of 2976 individuals with osteoarthritis, which found that insomnia (measured with the Insomnia Severity Index) was not independently associated with hip or knee replacement, nor did it increase the odds of joint replacement when combined with joint pain compared with joint pain alone.^23^ However, our study builds on this prior study by including a much larger sample, including over 100 000 individuals, specifically with hip and knee pain (ie, those most likely to progress to hip and knee replacement), and by including a much longer follow-up period (approximately 3 years vs 15 years in this study). Taken together, insomnia symptoms may contribute to earlier decision-making to undergo knee replacement. Beyond 4.7 years, the association attenuated, possibly due to time-varying effects and/or the selection of higher-risk individuals in the earlier period. However, these associations should be assessed in future studies, given that the assessment of insomnia in the UK Biobank did not address all diagnostic criteria.^12^

Caution should be used when comparing the magnitude of our findings with those of other studies, because of differences in study populations and the covariates used in modelling. However, the magnitude of the association we observed, between usually experiencing insomnia and undergoing knee replacement surgery, appears smaller than that of other established prognostic factors for knee replacement, such as high body mass index.^29^ Longitudinal studies suggest that weight reduction can lower the risk of joint replacement.^47^ In contrast, no studies have investigated whether improvements in insomnia reduce the risk of undergoing joint replacement. However, given the scale of the health and economic burden of knee osteoarthritis, even a small reduction in surgical demand could be meaningful. In England, the 2016/2017 adjusted estimate of 1-year hospitalisation costs for knee replacement was £7805, with 457 747 individuals undergoing the procedure between 2008–2017.^48^ The demand for total knee replacement in England and globally is projected to increase substantially over the coming decades.^7 8 49 50^ Thus, even if sleep interventions marginally reduce the need for knee replacement, particularly if they are low-cost and scalable, they may yield meaningful health system savings.

Contrary to our hypothesis, there was no association between symptoms of insomnia and joint replacement in those with hip pain. Other prognostic factors for symptomatic progression in hip osteoarthritis (ie, large acetabular bone marrow lesions, large femoral head bone marrow lesions, depressive symptoms and chronic widespread pain^51^) may have a more dominant role in driving the decision to undergo hip replacement. The differences in observed associations between insomnia and joint replacement for hip versus knee pain highlight that hip and knee osteoarthritis likely represent distinct clinical entities.^51^ Total joint replacements occurred in 13% of those with either knee pain and/or hip pain, and usual symptoms of insomnia were reported by 43% of our hip pain sample and 37% of the knee pain sample. These comparable rates of joint replacement events and presence of insomnia suggest that neither statistical power nor exposure misclassification is likely to explain the differential associations observed.

Individuals with knee osteoarthritis have greater engagement with passive pain coping strategies (eg, worrying, resting or withdrawing from activity) than those with hip osteoarthritis.^52^ This distinction is particularly relevant as passive coping is associated with greater disability in knee, but not hip osteoarthritis.^53^ Passive coping often involves cognitive and behavioural patterns such as rumination, inactivity and heightened emotional distress that impact sleep. These behavioural tendencies might amplify the impact of insomnia on pain in knee osteoarthritis, potentially in part explaining why an association between insomnia and joint replacement was observed for the knee but not the hip. The distinction may also reflect broader differences in care-seeking patterns. For example, individuals undergoing hip replacement tend to be more willing to undergo surgery,^5^ as supported by our data, with time to surgery being approximately 20% shorter in those with hip pain relative to knee pain. We speculate there would be less time for insomnia to worsen pain via central sensitisation, with central sensitisation being more widely reported in people with knee osteoarthritis; however, further research is needed to investigate its role in hip osteoarthritis.^54^

Our findings may stimulate future research. This includes whether a dose-response relationship exists such that greater severity of insomnia is associated with a greater risk of knee joint replacement. This is indirectly supported by our observation that ‘sometimes’ having symptoms of insomnia was not associated with knee replacement, while reporting ‘usual’ symptoms of insomnia was associated with undergoing knee replacement. Further studies are needed to assess whether, indeed, insomnia is causally related to progression to knee replacement for knee OA, and whether improving insomnia reduces the risk of knee replacement. Future research could also consider measures of sleep obtained using actigraphy with a focus on capturing objective data beyond self-reported symptoms of insomnia.

Strengths of this study include its large sample size, cohort study design with a median follow-up of over 13 years, and the inclusion of hip pain, given that hip osteoarthritis is often an underrepresented focus in osteoarthritis research relative to knee osteoarthritis. However, several limitations warrant consideration. Given the size of the estimated e-value and the potential for unobserved confounders (ie, covariates for which we had no data in our dataset), it is plausible that the observed association could be explained away by a confounding variable for which we were unable to adjust. Despite our prospective design and use of the E value to assess the impact of potential residual confounding, information bias may still be present. There are also several limitations related to our exposure variable, insomnia. It is possible that insomnia symptoms reported as ‘usually’ or ‘sometimes’ reflected transient rather than persistent experiences, which may have a weaker association with rates of joint replacement. Although sleep problems such as insomnia persist over a lifetime for many individuals,^55^ we only included insomnia reported at baseline due to substantial attrition in follow-up data (n=20 328 with hip and/or knee pain at the first reassessment). Additionally, while the sleep items captured key symptoms of insomnia, they did not assess all diagnostic criteria,^12 56^ such as related daytime impairments (eg, fatigue), limiting the precision of the exposure definition. This may affect comparisons to other studies on insomnia and the translation of our findings to clinical populations.^57^ Lastly, as symptoms of insomnia and joint pain were assessed only at baseline, the UK Biobank data do not permit causal inference regarding the impact of insomnia severity on joint pain. Future studies should capture all diagnostic criteria for insomnia and include longitudinal measures of insomnia and joint pain to assess whether insomnia causally relates to worsening joint pain and progression to joint replacement.

There were also potential limitations in how joint pain and the incidence of total joint replacements were captured in the UK Biobank. The UK Biobank only collects data on joint replacements within the public health system, so any joint replacements performed in the private sector are not accounted for in this study. We used a pragmatic approach by including participants with knee or hip pain over the past 3 months, rather than restricting the sample to those with a formal osteoarthritis diagnosis. This decision was driven by the substantially smaller sample size of those diagnosed with hip or knee osteoarthritis at baseline. Underreporting of osteoarthritis diagnoses in UK electronic health records^58^ could lead to misclassification bias. By including all individuals likely to have osteoarthritis-related pain, we aimed to maximise generalisability. However, particularly for those with hip pain, other diagnoses (eg, gluteal tendinopathy or femoroacetabular impingement) may have been the primary drivers of joint pain.^59 60^ Diagnoses other than osteoarthritis could differ in their associations with joint replacement, with osteoarthritis-related pain potentially more likely to progress to joint replacement than other causes of pain. Future replication in osteoarthritis-specific cohorts would strengthen understanding of how sleep disturbance contributes to illness progression (eg, pain) and progression to surgery.

Individuals with knee pain who reported usually experiencing insomnia were more likely to undergo total knee replacement surgery within, but not beyond, 4.7 years of follow-up. In contrast, individuals with hip pain and symptoms (‘usual’ or ‘sometimes’) of insomnia were no more likely to undergo total hip replacement surgery than those who did not experience insomnia symptoms. Targeting insomnia through interventions could form part of a holistic approach to managing chronic knee pain. However, further research is needed to determine whether managing insomnia can indeed reduce the risk of knee replacement.

## Supplementary material

10.1136/rmdopen-2025-006357 online supplemental file 1

## Data Availability

Data may be obtained from a third party and are not publicly available.
