# Supplementary material for: Insomnia and progression to total joint replacement in hip (41 737) and knee pain (81 958): a prospective UK biobank cohort study
Source: RMD Open. 2026 Jan 16;12(1):e006357. doi: 10.1136/rmdopen-2025-006357 (PMC12815097; doi:10.1136/rmdopen-2025-006357)
Supplement: online supplemental file 1 [file rmdopen-12-1-s001.docx]

**UK Biobank codes for total knee and hip replacement**

Knee replacements were identified with the codes "W40.1 Primary total prosthetic replacement of knee joint using cement", "W41.1 Primary total prosthetic replacement of knee joint not using cement”, and "W42.1 Primary total prosthetic replacement of knee joint NEC.” Hip replacements were identified with the codes “W37.1 Primary total prosthetic replacement of hip joint using cement", "W38.1 Primary total prosthetic replacement of hip joint not using cement", "W39.1 Primary total prosthetic replacement of hip joint NEC", and "W94.1 Primary hybrid prosthetic replacement of hip joint using cemented femoral component.”

**Directed acyclic graph**

**
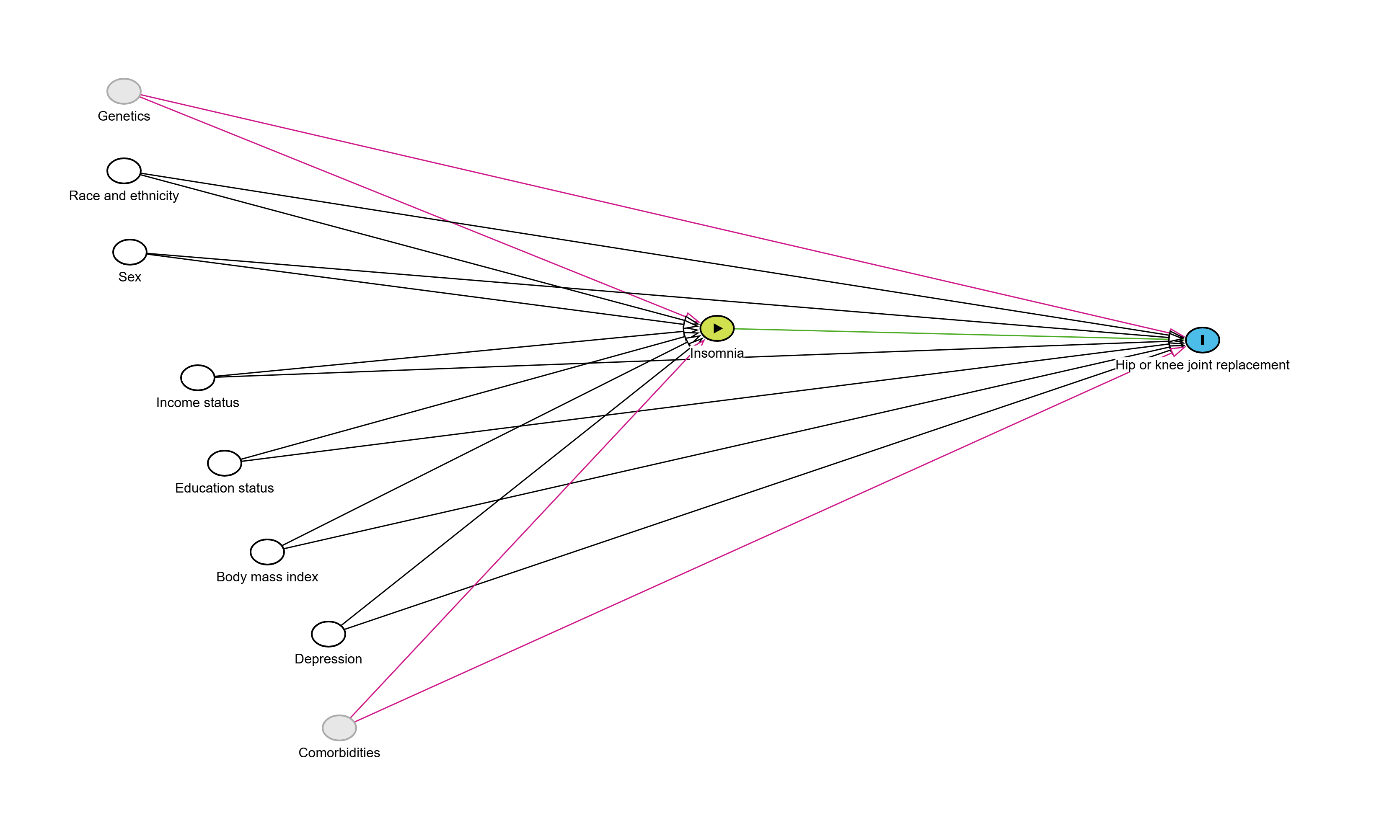
**

**Figure 1.** Directed acyclic graph. White circles indicate observed confounders; grey circles indicate unobserved confounders; green indicates exposure; blue indicates the outcome.

**Supplementary File 1. Covariate Selection**

The research term acknowledges that many of the proposed relationships are potentially bidirectional. We formed consensus on the following covariates by considering the weight of the evidence and our existing theoretical and clinical knowledge. Below, we highlight key studies that inform our understanding, but this is not intended to be a comprehensive summary of the available literature in this space.

Sex was chosen as a covariate because of evidence that it is associated with both osteoarthritis progression. A recent review found that females, compared with males, were more likely to experience a range of poorer outcomes, including osteoarthritis pain severity [30]. In hip and knee OA (GLA:D® Cohort), being female was associated with greater sleep disturbances [32]. A large cohort study (>100,000 participants) found that being overweight or obese was associated with an increased risk of knee replacement among individuals with knee osteoarthritis [31].

Higher body mass index is also associated with worse sleep quality, although this relationship is likely bidirectional—as shown in a prospective cohort study of 1,031 adults—and has not been established among people with osteoarthritis specifically [33]. After discussions among the research team, we opted for a conservative approach and included BMI as a covariate in our models, because we felt there was sufficient evidence and theoretical plausibility that BMI could confound any observed associations.

Associations between socioeconomic factors and the risk of joint replacement procedures are also reported [29, 34]. For instance, in a large population-based case-control study involving 104,055 total hip replacement patients, indicators of lower socioeconomic status, such as lower income and education, were associated with an increased risk of total hip replacement [34]. A systematic review (n=19 studies) and meta-analysis found that lower socio-economic status—which included measures of socio-economic status and proxies, such as income, neighbourhood area, and employment status—was associated with poorer measures of sleep quality (e.g., efficiency, latency, and fragmentation); in contrast, higher socio-economic status was associated with better sleep quality [35]. A narrative review of race and ethnicities found that most research had compared hip and knee osteoarthritis outcomes between African Americans and White Americans [29]. Summarising the evidence, it concluded that African Americans tended to experience a higher osteoarthritis burden when compared with White Americans. This included experiencing more severe pain, poorer physical function, and greater clinical progression of osteoarthritis (e.g., pain worsening). Sleep quality (device-measured and sleep report) is also associated with reported race and/or ethnicity. Among individuals with knee osteoarthritis (96 African Americans and 128 non-Hispanic Whites), African Americans tended to experience more awake time at night, higher sleep fragmentation, and lower sleep efficiency compared with non-Hispanic Whites [36]—sleep was measured via a wrist-worn accelerometer.

The research team initially did not include depression in the Cox Regression Models as a covariate because of concerns about its temporal relationship with the exposure and outcome. In response to reviewers' comments and further consideration of exploratory literature suggesting a possible role of depression in exacerbating joint pain [37], and a plausible association between depression and insomnia (though no evidence was found in osteoarthritis specifically), the research team agreed it could confound any observed associations. We thus included it in separate models for hip and knee pain in addition to the above-mentioned covariates.

**Covariate handling**

| **Supplementary Table 1.** Collapsing of covariate variables | | |
| --- | --- | --- |
| **Created variable** | **Created levels of variable*** | **UK Biobank codes/values** |
| Body mass index^#^ |  |  |
|  | Underweight | 18.5 |
|  | Healthy weight | 18.5 to <25 |
|  | Pre-obesity | 25 to <30 |
|  | Obesity | ≥ 30 |
| Education |  |  |
|  | No Degree or Professional Qualification | “A levels/AS levels or equivalent”,” O levels/GSCEs or equivalent”, “CSEs or equivalent”, “NVQ or HND or HNC or equivalent |
|  | Degree or Professional Qualification | “College or University Degree”, “Other professional qualifications eg: nursing, teaching” |
|  | Prefer not to say or none of the above | “Prefer no to Say”, “None of the above” |
|  | Unknown | Missing data. |
| Ethnicity |  |  |
|  | Asian or of part Asian background | "Indian", "Pakistani", "Asian or Asian British", "Bangladeshi", "Any other Asian background", "White and Asian", "Chinese" |
|  | British, Irish, and/or White | "White", "British", "Irish", "Any other white background" |
|  | African, Black, Caribbean, or with part Black background | African", "Caribbean", "White and Black African", "White and Black Caribbean", "Black or Black British", "Any other Black background" |
|  | Other ethnic group | "Mixed", "Any other mixed background", "Other ethnic group” |
|  | Mixed background | ”Mixed" |
|  | Do not know or Prefer not to say | "Do not know", "Prefer not to answer" |
| *factor levels used in adjusted multivariable Cox regression models  ^#^height and weight measurements made at initial assessment centre visit | | |

**Sensitivity analysis: Risk ratio for confounder-outcome relationship**


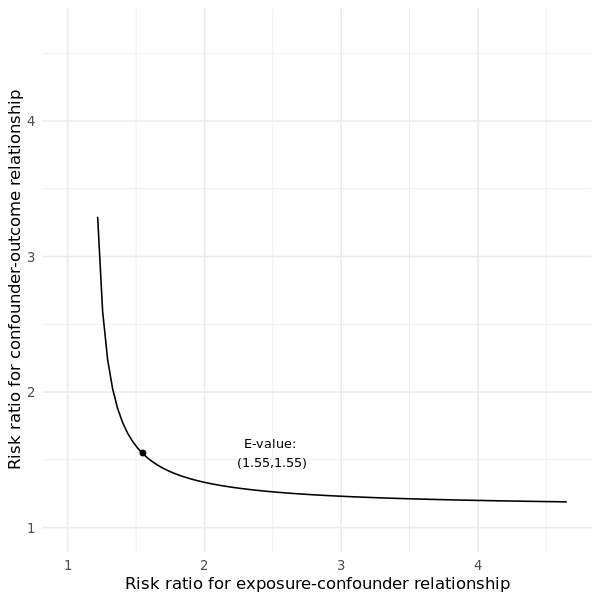


**Figure 2**. E value for the association between usually experiencing insomnia and an incident of total knee joint replacement.


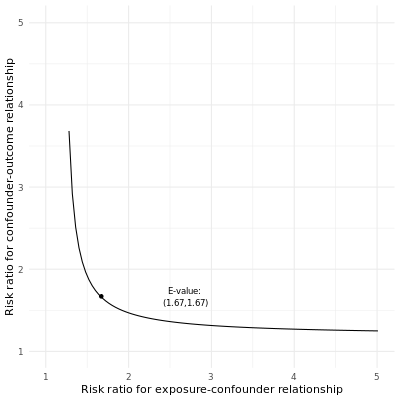


**Figure 3**. Sensitivity analysis: E value for the adjusted association between usually experiencing insomnia and an incident of total knee joint replacement, including an additional covariate of depression

**Table 2.** Sensitivity analyses of Cox models for the association of symptoms of insomnia and incident total knee replacement, imputing all missing country codes as Wales (n=81,958 participants)

|  | **Time period 1: 0-4.7 years** | | **Time period 2: >4.7 years** | |
| --- | --- | --- | --- | --- |
| **Signs of insomnia** | HR (95% CI)^*^ |  | HR (95% CI)^*^ |  |
| **Sometimes** | 1.01 (0.92 to 1.11) |  | 1.01 (0.95 to 1.08) |  |
| **Usually** | 1.14 (1.04 to 1.25) |  | 0.99 (0.93 to 1.06) |  |
| HR: hazard ratio  Adjusted cox proportional hazards models model estimating associations between signs of insomnia and total knee replacement with ‘never’ signs of insomnia as referent. Time groups T1 and T2 included as interaction term.  ^*^Adjusted for sex, body mass index, ethnicity, education, and Townsend Deprivation Index. | | | | |

| **Table 3** Unadjusted and adjusted associations between symptoms of insomnia and incident total joint replacement in people without co-existing hip and knee pain | | | | | | | | | |
| --- | --- | --- | --- | --- | --- | --- | --- | --- | --- |
|  | **Unadjusted analysis** | | |  | **Adjusted analysis*** | | | |  |
|  | **Knee pain**  (n = 63,316) | | **Hip pain** (n=23,095) |  | **Knee pain**  (n = 63,316) | | **Hip pain**  (n=23,095) |  |  |
| **Symptoms of insomnia** | Time period 1:  < 4·7 years  HR (95% CI) | Time period 2: > 4·7 years  HR (95% CI) | HR (95% CI) |  | Time period 1:  < 4·7 years HR (95% CI) | Time period 2:  > 4·7 years  HR (95% CI) | HR (95% CI) |  |  |
| Sometimes | 1·04 (0·94 to 1·15) | 1·06 (0·99 to 1·14) | 0·97 (0·88 to 1·07) |  | 1·01 (0·91 to 1·12) | 1·03 (0·96 to 1·10) | 0.98 (0·88 to 1·08) |  |  |
| Usual | 1·24 (1·12 to 1·37) | 1·09 (1.01 to 1·17) | 0·94 (0·84 to 1·04) |  | 1·16 (1·05 to 1·29) | 1·02 (0·95 to 1·10) | 0.95 (0·86 to 1·06) |  |  |
| Never | REF | REF | REF |  | REF | REF | REF |  |  |
| CI: confidence interval; HR: Hazard ratio; REF: reference  Adjusted Cox proportional hazards models estimating associations between sleeplessness and total knee replacement.  *Adjusted for sex, body mass index, ethnicity, education, and Townsend Deprivation Index | | | | | | | |  |  |
